# Paravalvular Leak in Transcatheter Aortic Valve Implantation: A Review of Current Challenges and Future Directions

**DOI:** 10.3390/jcdd12040125

**Published:** 2025-03-31

**Authors:** Andreas Synetos, Nikolaos Ktenopoulos, Odysseas Katsaros, Konstantina Vlasopoulou, Maria Drakopoulou, Leonidas Koliastasis, Ioannis Kachrimanidis, Anastasios Apostolos, Sotirios Tsalamandris, George Latsios, Konstantinos Toutouzas, Ioannis Patrikios, Constantinos Tsioufis

**Affiliations:** 1 First Department of Cardiology, National and Kapodistrian University of Athens, Hippokration General Hospital of Athens, 11527 Athens, Greece; nikosktenop@gmail.com (N.K.); odykatsaros@gmail.com (O.K.); vlasopouloukon@gmail.com (K.V.); mdrakopoulou@hotmail.com (M.D.); lkoliastasis@gmail.com (L.K.); iskachrimanidis@gmail.com (I.K.); stsalamandris@hotmail.com (S.T.); glatsios@gmail.com (G.L.);; 2 Medical School, European University of Cyprus, 2404 Egkomi, Cyprus

**Keywords:** transcatheter aortic valve implantation, paravalvular leak, aortic stenosis, paravalvular regurgitation

## Abstract

Transcatheter aortic valve implantation (TAVI) has emerged as a revolutionary therapeutic modality for the management of severe aortic stenosis (AS), particularly in patients who are at high or prohibitive risk for surgical aortic valve replacement (SAVR). Over the past decade, extensive clinical evidence has expanded the indications for TAVI to include intermediate- and low-risk populations, which usually represent a population of younger age, in which the most common complications of TAVI, including paravalvular leak (PVL) and pacemaker implantation, should be avoided. This review focuses on the incidence and predictors of PVL in various types of TAVI implantation, its clinical implication, and the prevention strategies to tackle this complication.

## 1. Introduction

Transcatheter aortic valve implantation (TAVI) has emerged as a revolutionary therapeutic modality for the management of severe aortic stenosis (AS), particularly in patients who are at high or prohibitive risk for surgical aortic valve replacement (SAVR) [1,2,3,4,5]. Over the past decade, extensive clinical evidence has expanded the indications for TAVI to include intermediate- and low-risk populations [6,7,8]. This paradigm shift underscores the significant advantages of TAVI, including shorter recovery times, reduced procedural morbidity, and comparable or superior survival outcomes relative to SAVR. Alongside, in many developed nations, TAVI has now surpassed SAVR as the most commonly performed procedure for aortic valve replacement [9].

Despite these advancements, TAVI is not without limitations. One of the most persistent and clinically significant complications is paravalvular leak (PVL), defined as regurgitant blood flow through gaps between the prosthetic valve and the native annulus [10,11]. PVL remains one of the most clinically significant complications following TAVI. Its incidence has dramatically decreased over the past decade due to advancements in valve technology, enhanced pre-procedural imaging, and refined deployment techniques. Early-generation transcatheter heart valves (THVs) were associated with alarmingly high rates of PVL, with moderate to severe PVL observed in up to 40% of patients [12,13]. On the other hand, mild PVL remains prevalent, occurring in approximately 20–30% of the cases, even with the latest THV iterations. However, continuous improvements in THV design, including the incorporation of external skirts and enhanced deployment precision, aim to reduce PVL rates [13].

PVL severity is categorized as mild, moderate, or severe based on echocardiographic and clinical criteria [13]. Moderate-to-severe PVL has been associated with an elevated risk of mortality, heart failure, and rehospitalization, while even mild PVL may contribute to adverse long-term outcomes, including left ventricular remodeling and chronic heart failure [10]. The clinical implications of PVL are profound and multifaceted. Moreover, PVL adversely affects hemodynamics, leading to left ventricular remodeling, reduced cardiac efficiency, and a heightened need for reintervention. These sequelae have far-reaching implications for patient quality of life and healthcare resource utilization [11,14,15].

Improvements in imaging modalities, such as 3D computed tomography (CT) and transesophageal echocardiography (TEE), have significantly enhanced pre-procedural planning and intra-procedural assessment, enabling better prediction and prevention of PVL [14,16]. Additionally, newer-generation THVs with advanced sealing mechanisms have been instrumental in reducing PVL rates. However, despite these advancements, the presence of even trace PVL remains a clinical concern, particularly as TAVI expands into younger, low-risk populations with longer life expectancies [17]. Management strategies for PVL vary based on the severity of the leak and its clinical impact [13]. Percutaneous closure techniques using vascular plugs, post-deployment balloon dilation, and, in some cases, valve-in-valve implantation have shown promise in reducing PVL severity and improving outcomes [1,18].

This review aims to provide a comprehensive examination of PVL in the context of TAVI, with a focus on its incidence, predictors, mechanisms, diagnostic modalities, and management strategies. Additionally, the review explores emerging technologies and procedural innovations aimed at mitigating PVL risk to ensure optimal outcomes for patients undergoing TAVI. Addressing PVL is essential for advancing TAVI into broader patient populations and improving long-term outcomes.

## 2. Incidence and Predictors of Paravalvular Leak

### 2.1. Epidemiology of PVL

The incidence of PVL has markedly decreased over the past decade due to advancements in transcatheter heart valve (THV) technology and procedural techniques. Early-generation devices, such as the Sapien XT and CoreValve, exhibited PVL rates of up to 40%, particularly in patients with complex annular geometries or heavy calcification [6,19,20,21]. The introduction of newer-generation devices, including Sapien 3 and Evolut Pro, with enhanced sealing mechanisms and conformability, has reduced the prevalence of moderate-to-severe PVL to below 5%. However, mild PVL persists in approximately 20–30% of cases and has been increasingly associated with adverse long-term outcomes [13].

Geographical and institutional variability in PVL rates also reflects differences in procedural volume and operator expertise. High-volume centers with experienced operators consistently report lower PVL rates, emphasizing the importance of standardization and best practices in procedural training [15].

### 2.2. Prevalence and Prognosis of Paravalvular Regurgitation

The incidence of moderate-to-severe PVL following TAVI has significantly declined due to the advancements in valve design, implantation techniques, and operator expertise [22]. Nonetheless, recent data on the latest-generation THVs implanted in low-risk individuals indicate that moderate-to-severe PVL remains present in 0.8% of patients with balloon-expandable valves (BEV) and 3.4% of those with self-expandable valves (SEV) at 30 days. In contrast, mild PVL remains prevalent, occurring in approximately 29% of cases with BEVs and 36% with SEVs at 30 days [13]. Alongside, PVL is well-established as a significant prognostic marker, consistently associated with increased mortality. However, the clinical significance of mild PVL is more controversial, with conflicting evidence regarding its impact on survival. These discrepancies may be attributed to variations in patient populations, procedural risk levels, and the grading systems used to quantify PVL. For instance, the PARTNER trial for high-risk patients, which utilized a three-class grading system, demonstrated that mild PVL was associated with increased mortality compared to none or trace PVL (HR: 1.37; 95% CI: 1.14–1.90). In contrast, the PARTNER 2 trial for intermediate-risk patients, which adopted a five-class grading scheme, found no significant association between mild PVL and mortality (HR: 1.09; 95% CI: 0.84–1.41) [7,8].

Similarly, the Swiss TAVI registry further emphasizes the grading scheme’s influence on prognostic assessments. Using a three-class grading scheme, mild PVL was associated with an increased mortality risk at five years (HR: 1.56; 95% CI: 1.20–2.02). However, when a five-class grading scheme was applied, only mild-to-moderate PVL, and not mild PVL, was linked to higher mortality [23].

Nonetheless, one meta-analysis of 25 primarily non-comparative studies pooling over 21,000 patients found that mild PVL was associated with a 26% increase in all-cause mortality and a 28% increase in cardiovascular mortality compared to none or trace PVL [10]. More recently, an analysis of Kaplan–Meier-derived individual patient data from 38 predominantly non-randomized studies encompassing over 25,000 patients revealed that any degree of PVL, including mild PVL, was associated with increased risks of all-cause mortality, cardiovascular mortality, and rehospitalization [24].

It is essential to consider the heterogeneity included in these meta-analyses, particularly regarding the timing of PVL assessment and the grading systems employed. Additionally, many studies involved unadjusted cohorts, introducing potential confounding factors that may influence outcomes. These limitations underscore the need for standardized assessment frameworks and robust adjustment for confounders in future research to better delineate the true prognostic impact of mild PVL [25].

### 2.3. Predictors of PVL

#### 2.3.1. Patient-Specific Predictors

1. Body Mass Index (BMI): Low BMI is associated with suboptimal valve seating, likely due to reduced myocardial support and altered anatomical dynamics.
2. Left Ventricular Ejection Fraction (LVEF): Reduced LVEF impairs hemodynamic efficiency and amplifies the adverse effects of residual PVL.
3. Annular Size and Shape: Extreme annular dimensions (small or large) and non-circular annular shapes increase PVL risk due to challenges in device selection and sealing [25].

#### 2.3.2. Anatomical Predictors

1. Calcification Distribution: Heavy and asymmetric calcification within the aortic annulus or left ventricular outflow tract (LVOT) disrupts valve seating, leading to PVL.
2. Bicuspid Aortic Valves: Bicuspid anatomy is associated with higher PVL rates due to asymmetrical annulus and calcification [15].

#### 2.3.3. Device-Specific Predictors

**Figure 1:**
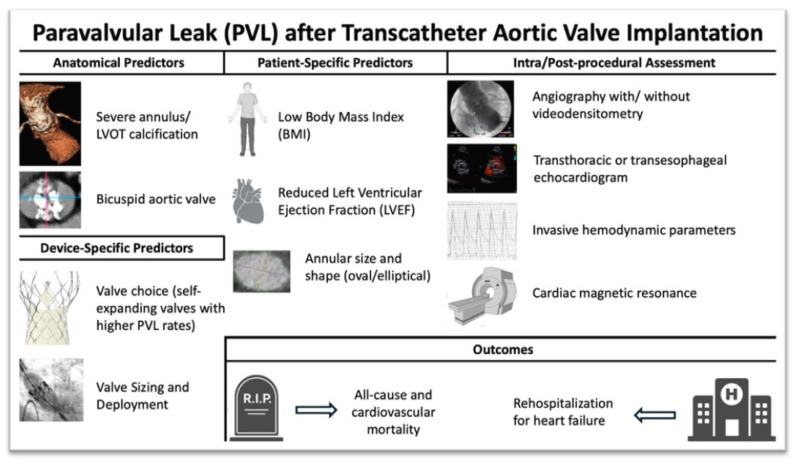
Predictors, intra- and post-procedural assessment, and outcomes of paravalvular regurgitation after transcatheter aortic valve implantation (TAVI).

1. Valve Type: Self-expanding valves, such as Evolut Pro, exhibit higher PVL rates compared to balloon-expandable valves like Sapien 3, reflecting differences in radial force and sealing capabilities [2,26].
2. Valve Sizing and Deployment: Improper valve sizing or suboptimal deployment depth increases PVL risk. Advanced imaging techniques, such as 3D computed tomography (3D-CT), are essential for accurate pre-procedural planning [17] (Figure 1).

### 2.4. Multifactorial Interactions and Predictive Models

PVL risk arises from the interplay of patient-specific, anatomical, and device-related factors. Emerging predictive models integrating clinical, imaging, and procedural data offer promise for personalized risk stratification and procedural optimization. Despite significant progress, current predictive models lack integration of advanced imaging and computational fluid dynamics. Machine learning algorithms and real-time biomechanical simulations may enhance PVL prediction and prevention when implemented [13,27].

While advancements in valve design and procedural techniques have substantially reduced the incidence of moderate-to-severe PVL, even mild PVL remains a clinically significant challenge with potential long-term implications. Understanding the complex interplay of predictors is essential for optimizing patient outcomes. As TAVI continues to expand into younger, lower-risk populations, addressing PVL will require further innovations in valve technology, advanced imaging modalities, and procedural standardization. Future research should focus on integrating novel technologies, such as machine learning and computational modeling, to refine risk stratification and ensure the long-term success of this transformative therapeutic intervention.

## 3. Mechanisms and Pathophysiology

During the transcatheter implantation of a prosthetic aortic valve, in contrast to surgical aortic valve replacement, the native aortic valve is not removed but crushed. A residual PVL represents the consequence of the existence of a gap between the transcatheter heart valve (THV) and the native surrounding structures, including the aortic valve leaflets, the aortic annulus, and the left ventricular outflow tract (LVOT). Several PVL mechanisms have been described in the literature. Calcification of the native aortic valve and asymmetrical calcium distribution impeding proper sealing, undersizing of the THV for the native annular size, and malpositioning of the THV (too high or low in the annulus) are the most recognized [28,29,30].

Although the calcium of the native annulus complex provides an anchorage to the THV’s stent frame, a very calcified aortic valve may result in stent under-deployment and neo-leaflets malapposition. The calcification severity, its asymmetrical distribution, and the location of the calcium (leaflets, commissures, annulus, or LVOT) have been identified in several studies as predictors for significant PVL [28,29,30,31,32,33,34,35,36]. Aortic valve calcium score (AVCS), measured on preoperative contrast-enhanced multislice computed tomography (MSCT), is significantly associated with device success and PVL [28,37,38]. Quantitatively, although without optimal sensitivity and specificity, a calcium volume threshold of >1000 mm3 has been associated with mild PVL or greater [34]. Additionally, sex-specific AVCS cutoff values of 4070 in men and 2341 in women were shown to be independent predictors of moderate PVL [39]. Regarding the calcium distribution, the presence of bulky calcifications, the asymmetrical distribution, predominantly in one cusp, and the calcification of the device landing zone comprising the annulus and LVOT are powerful independent predictors of residual PVL [32,33,37,38,40,41,42]. Notably, the impact of the valve calcification might be different according to the THV. The disadvantageous effect appears to be more pronounced with the use of self-expanding valves [34,37,43,44].

Several studies have suggested that undersizing of the TAVI THV results in greater degrees of PVL as it fails to generate a good apposition on the native annulus [28,29,30,36,45,46]. The annular eccentricity has been well-documented and has been implicated as a predictor of PVL [43,47,48]. Specifically, an eccentricity index of >0.25, calculated as 1 minus the ratio of minimal to maximal annulus diameter by MSCT, was shown as an independent predictor of significant PVL [47]. This noncircular geometry results in underestimation of the aortic annulus when using two-dimensional (2D) echocardiography. Therefore, during pre-procedural planning, three-dimensional (3D) measurements by MSCT are considered the gold standard for accurate annulus sizing [28,49]. The accuracy of 3D transesophageal echocardiography (TEE) for annular sizing and prediction of PVL severity has also been demonstrated [28,50]. An additional factor that has been shown to predict moderate–severe PVL due to undersizing is a small, achieved balloon size during pre-implantation valvuloplasty [29]. It has been suggested that oversizing of the THV relative to the MSCT mean annular diameter by at least 1 mm and annular area by at least 10% has a significantly reduced risk of moderate or severe PVL, being less influenced by the eccentricity of the annulus [35,51]. However, in the presence of moderate–severe subannular/LVOT calcification, aggressive THV oversizing (≥20%) has been associated with an increased risk of aortic root rupture during TAVI with balloon-expandable THVs [52,53,54,55]. In this setting, in carefully selected patients, a strategy of deliberate under-expansion (balloon-filling volume reduced <10%) with ad hoc post-dilation, if necessary, can be used to reduce the risk of annular injury without compromising valve performance [52,56,57].

The implantation depth, either excessively high or low, is associated with residual PVL [28,29,30]. An exceedingly high implantation results in inadequate apposition of the frame and anchoring of the THV, leading to inadequate sealing and consequently PVL. Very low implantation results in PVL through the stent and above the skirt of the THV. The unequal geometry of the self-expanding CoreValve with a narrow midsection contributes further to PVL due to inadequate sealing if malpositioned [30]. Sherif et al. showed that the chance of significant PVL is the lowest when the depth of the device in relation to the noncoronary cusp is about 10 mm [58]. Moreover, a greater angle of the LVOT to the ascending aorta was associated with a greater chance of significant PVL [58,59].

The occurrence of PVL after TAVI has a negative impact on left ventricular (LV) function and hemodynamics. The LV hypertrophy that accompanies severe aortic valve stenosis results in impaired relaxation, increased stiffness, decreased compliance, myocardial fibrosis, diastolic dysfunction, and elevated LV end-diastolic pressure. In this setting, the regurgitation volume of the PVL, behaving as acute aortic regurgitation, contributes to elevation of the already increased end-diastolic pressures of a hypertrophied ventricle not adapted to volume overload. Thus, even mild PVL can have detrimental hemodynamic consequences, possibly affecting survival [60,61].

## 4. Diagnosis

### 4.1. Echocardiography

A variety of diagnostic tests should be performed to determine the presence and degree of PVL. The Valve Academic Research Consortium-3 (VARC-3) recommends the use of echocardiography as the first-line imaging modality for the assessment of PVL [62]. Multiple PVL jets arising from several sites in the paravalvular space with irregular shapes and directions are frequently detected post-TAVI, creating challenges in the assessment of the regurgitation severity. Thus, the echocardiographic assessment of PVL requires several modalities, including transthoracic (TTE) and transesophageal echocardiography (TEE), utilizing 2D, 3D, and Doppler echocardiographic techniques. TTE is easily available, inexpensive, and noninvasive, and it is usually the initial imaging modality used to assess patients with TAVI [28,63,64,65]. This is also reinforced by the trend towards minimally invasive TAVR under local anesthesia or conscious sedation [66,67]. A recent study supports the routine use of intra-procedural TTE immediately after TAVI deployment as it allows operators to perform post-balloon dilatation with improvement in early echocardiographic results [68]. Parasternal long- and short-axis views as well as apical and off-axis views are often needed to ensure that all jets are identified. However, because of acoustic shadowing from the THV, posterior PVL jets may be masked with TTE, while anterior jets may not be displayed during TEE. Consequently, TTE and TEE are complementary in this regard to detect all sites of PVL. Although TEE is invasive, requires conscious sedation or anesthesia, and the quality of image acquisition and interpretation depends on the skills of the operator, it provides better spatial resolution and image quality. To override the acoustic shadow-driven limitation of anterior jet visualization by TEE, adjustment of the imaging angle or depth may allow more extensive visualization. Thus, trans-gastric views may be obtained, considering though that the Doppler angulation may not be optimal in this view [28,63,64,65]. In addition, 3D echocardiography plays a significant role in determining the precise location and size of the PVL with the limitations of reduced temporal and spatial resolution as well as acoustic shadow and echocardiographic dropout artifacts [63,67,69,70].

The initial assessment of PVL includes evaluation of the prosthetic valve structural integrity. The stability and motion of the sewing ring, or any abnormal space between the sewing ring and native annulus, may be the first indicators of valvular dehiscence and PVL. A rocking motion implies a large dehiscence, 40% to 90% of the sewing ring [63,65]. The evaluation of PVL should be an integrative approach using several qualitative, semi-quantitative and quantitative Doppler measurements, similarly to native or prosthetic valve regurgitation [28,63,64,65]. Color Doppler evaluation of the PVL jet requires visualization of the flow convergence, vena contracta (VC), and proximal jet extension into the LVOT and left ventricle. Acoustic reverberation and shadowing from the THV may impair visualization of the flow convergence and VC regions or assessment of the jet width in the LVOT. On the contrary, semiquantitative and quantitative spectral Doppler methods for grading regurgitation severity are not affected by the prosthetic valve [64].

The European Association of Cardiovascular Imaging and the American Society of Echocardiography recommend a three-class grading scheme (mild, moderate, severe) to report the severity of PVL [28,64,65]. However, an expanded five-class grading scheme has been proposed and is frequently used in clinical practice. This scheme divides mild PVL into mild and mild-moderate PVL and moderate PVL into moderate and moderate-severe PVL. In this scheme, no PVL and trace PVL could be combined into grade 0 [62,71]. The clinical importance of this extended scheme emerged in a recent study when, after applying the five-class grading scheme, only mild-to-moderate PVL was associated with an increased risk of mortality, in contrast to mild PVL [72]. Table 1 summarizes the three- and five-class grading schemes with the suggested categorization of the qualitative, semi-quantitative, and quantitative parameters. If there is a discrepancy or inconsistency among parameters, then explanations from image quality, technical, and physiological factors should be identified. If consensus grading cannot be determined, cardiovascular magnetic resonance (CMR) or cardiac computed tomography (CCT) are likely required to identify the mechanism and grade the severity of regurgitation [64].

### 4.2. Cardiac Computed Tomography (CCT)

CCT is predominantly used in preprocedural planning in patients undergoing TAVI as it can provide accurate measurements of the annular size and shape as well as the location and degree of annular calcification, decreasing the risk of PVL [28,37,38,49]. Postprocedural CCT is a useful adjunct imaging modality when TTE and TEE are inconclusive in determining the grade or location of PVL, especially due to excessive acoustic shadowing in cases with severe calcification. It can also be utilized as an alternative in patients with significantly limited echocardiographic images. PVL is depicted as a contrast material–filled channel in the paravalvular region that connects the aorta and the LVOT. Ring dehiscence may be appreciated by a rocking displacement of the valve ring on dynamic CT reconstructions. Limitations of CCT are the need for iodine contrast medium and ionizing radiation and poor temporal resolution in patients with rapid and irregular heart rates, alongside its inability to assess flow and hemodynamics [63,64,65].

### 4.3. Cardiovascular Magnetic Resonance (CMR)

In cases of inconclusive findings of TTE and/or TEE or discordance between the echocardiographic PVL grading and patient’s symptoms and/or degree of LV dilatation or dysfunction, CMR may help to corroborate the severity of regurgitation [28,63,64,73,74]. CMR’s advantage over echocardiography is providing absolute regurgitant volumes and fractions irrespective of the number and eccentricity of the regurgitant jets. This is accomplished via phase-contrast imaging. In-plane phase-contrast imaging can help define trans- or paravalvular regurgitation using three-chamber and aorta coronal views, whereas through-plane phase-contrast imaging perpendicular to the aortic wall immediately above the prosthetic valve can facilitate direct measurement of both antegrade and retrograde flow. This allows the direct measurement of forward stroke volume and regurgitant volume and subsequent calculation of regurgitant fraction. The suggested CMR regurgitant fraction cutoffs are <30% for mild, 30% to 50% for moderate, and ≥50% for severe PVL [28,62]. The main limitation of the phase-contrast technique is that reverse flow by CMR includes coronary flow in addition to the regurgitation volume, resulting in overestimating PVL. Alternatively, the difference between forward aortic and net pulmonary flow can be used. Furthermore, the presence of holodiastolic flow reversal in the descending aorta has excellent sensitivity and specificity for identifying severe regurgitation. The anatomic regurgitant orifice area can also be obtained from the cine CMR acquisitions of the valve [28,63,64]. Emerging techniques in the field of flow assessment for accurate quantification of PVL after TAVI using CMR are 2D multi-velocity encoding (venc) flow mapping and 4D flow mapping. The fast acquisition of the 2D multi-venc sequence and the free-breathing acquisition with retrospective plane selection of the 4D flow sequence provide useful advantages in clinical practice [75]. Limitations of CMR include arrhythmias, inability of breath-holding, claustrophobia, valve-related artifacts, and metallic implants.

Studies comparing echocardiography with CMR in assessing paravalvular regurgitation have shown an underestimation of regurgitant volumes using TTE and TEE [76,77]. In a recent meta-analysis assessing aortic regurgitation (AR) after TAVI, despite the reported significant discordance between TTE and CMR, TTE showed sufficient ability to discriminate moderate or severe AR from mild or none [74]. Other advantages of CMR are the high reproducibility of measurements and the excellent intra- and inter-observer agreement, being superior to TTE in this regard [28,70].

### 4.4. Angiography and Hemodynamic Assessment

Aortic root angiography by an adjunct administration of contrast agent from a pigtail catheter placed in the ascending aorta just above the newly implanted THV can be used for intra-procedural semiquantitative assessment of AR severity. AR is classified by visual grading according to the Sellers four-class grading scheme [78]. This is usually performed during the TAVI procedure when AR is detected by continuous Doppler in order to further evaluate its severity. The limitations of this technique are the low reproducibility of classification by visual estimation, no good correlation with quantitative assessment, no reliable distinction of central from paravalvular regurgitation, dependence on technical factors such as the intensity and projection of fluoroscopy, the position of the pigtail catheter, the amount and speed of contrast injection, the possible projection of the descending aorta over the LV causing inaccuracy of grading, and the adjunct administration of contrast agent [28,63,71].

More recently, quantitative videodensitometry has been proposed as a more reproducible measure of AR following TAVI by comparing the density of the contrast agent in the aorta with that in the ventricle using time–density curves generated in the LVOT and in the aortic root. The regurgitant fraction is then calculated by dividing the area under the curve (AUC) of the LVOT by the AUC of the aortic root. The regurgitant fraction by this technique correlates well with the CMR regurgitation fraction [79,80].

Several quantitative invasive hemodynamic parameters have been proposed to assess the severity of PVL and the prognosis immediately post-TAVI (Table 2). These parameters use the hemodynamic tracings obtained during the TAVI procedure; hence, no additional procedural time or contrast agent is required [28,71,81,82,83,84,85,86,87,88,89,90]. The assumption is that the difference between aortic and LV diastolic pressures decreases with increasing severity of AR. Their limitations are the heart rate dependence, the inability to distinguish valvular from paravalvular regurgitation, and the dependence on ventricular and aortic compliance and on aortic stiffness [28,71].

## 5. Clinical Implications

### 5.1. Outcomes

The full impact of PVL on outcomes after TAVI is not yet completely understood, and the threshold for what constitutes clinically significant PVL remains uncertain. While earlier reports suggested that mild paravalvular regurgitation (PVR) is commonly observed after TAVI and typically results in a benign prognosis, other studies have indicated that PVL may be linked to higher late mortality rates [91] (Figure 1). Recently, a large observational study involving over 1500 patients found that any degree of PVL after TAVI negatively impacted both overall survival and functional status. A meta-analysis of 17 observational studies, including approximately 15,000 patients, confirmed the negative effects of significant PVR on outcomes, revealing that moderate or worse post-TAVI PVL was associated with a twofold increase in all-cause mortality [23]. A meta-analysis of 25 studies involving over 21,000 patients demonstrated a 26% increase in all-cause mortality and a 28% increase in cardiovascular mortality among patients with mild PVL compared to those without PVL [92]. Furthermore, major registries and trials emphasize the prognostic significance of PVL severity. For instance, in the PARTNER trial, mild PVL was associated with a higher risk of mortality compared to no or trace PVL (HR: 1.37; 95% CI: 1.14–1.90) [92]. However, in the PARTNER 2 trial, using a five-class grading system, mild PVL did not show a significant increase in mortality risk (HR: 1.09; 95% CI: 0.84–1.41) [93]. Given the limited data on the incidence and predictors of PVL following TAVI, further research is needed to identify both patient-related and prosthesis-related factors that could potentially be modified to reduce PVL occurrence.

### 5.2. Comparison of Valve Types

PVL incidence varies with the type of transcatheter heart valve (THV) used. Self-expanding valves, such as the Medtronic Evolut Pro, exhibit higher PVL rates than balloon-expandable valves like the Edwards SAPIEN 3. This disparity is attributed to differences in radial force distribution, sealing mechanisms, and annular conformity. Newer-generation THVs with enhanced sealing skirts have significantly reduced moderate to severe PVL rates to below 5%. Studies comparing THV types, including the REPRISE III trial, demonstrated lower PVL rates with the Lotus valve compared to the CoreValve. However, mild PVL remains prevalent across all valve platforms, with rates of 20–30% depending on the device [94].

## 6. Prevention and Treatment

### 6.1. Prevention Strategies

Effective prevention of PVL begins with meticulous pre-procedural planning. Advanced imaging modalities, such as 3D CT and transesophageal echocardiography (TEE), enable precise valve sizing and identification of anatomical challenges, such as heavy annular calcification or non-circular annular shapes. Detailed measurements of the annulus, left ventricular outflow tract (LVOT), and coronary heights are essential to minimize PVL risk. Innovative valve designs have incorporated features to enhance sealing. External skirts, adaptive sealing mechanisms, and repositionable valves, such as the Lotus and Evolut Pro, have demonstrated improved outcomes, particularly in addressing complex anatomies [94].

### 6.2. Expanded Insights and Data

Recent studies have further emphasized the significance of PVL management in TAVI:

- Percutaneous Closure Success: In a multicenter registry involving patients with severe PVL post-TAVI, closure using Amplatzer Vascular Plugs III achieved a high procedural success rate, significantly improving functional outcomes and reducing hospitalizations for heart failure [95].
- Innovative Imaging Techniques: Arterio-arterial rail methods have enhanced closure success rates by enabling precise plug positioning, minimizing procedural risk [96].
- Valve-Specific Data: The Boston Lotus Valve and Medtronic Evolut R have demonstrated lower PVL rates in head-to-head comparisons, showcasing the impact of novel design features such as adaptive sealing and recapturability [97,98,99].

### 6.3. Treatment Options for PVL

1. Balloon Post-Dilation: In cases of acute leaks, repeated balloon post-dilation (PD) of an under-expanded valve may aid in achieving better expansion and a more effective seal, thereby reducing PVL [100]. Notably, an oversized balloon is recommended to fully expand the implanted valve. Several studies have demonstrated that balloon PD is both safe and effective in reducing significant regurgitation in the majority of patients, regardless of whether they have self-expandable or balloon-expandable prostheses. However, balloon PD may not always reduce PVL in certain patients, and it has been associated with an increased risk of stroke or transient ischemic attack [13]. Post-dilation is effective in reducing PVL severity by improving valve apposition, but this technique carries potential risks, including annular rupture and embolization. Therefore, the decision to perform post-dilation should be guided by intra-procedural imaging [94].
2. Snare Technique: Improper implantation depth has been linked to an increased risk of developing PVL after TAVI. With the advent of newer-generation devices, it is now possible to reposition the prosthesis before final deployment if the operator is dissatisfied with its position. This technique has been shown to reduce the occurrence of PVL [98,101]. Additionally, a snare loop-assisted device has proven effective in repositioning a prosthesis that is implanted too deeply, as it allows the operator to pull the device upward by attaching a snare to one of the frame loops [102]. However, snaring should be performed with caution in patients with heavily calcified valves, as calcium deposits may become dislodged and lead to complications [103].
3. Valve-in-Valve Implantation: Implanting a second valve may be an effective option when balloon post-dilation (PD) or other techniques are not suitable or fail to improve PVL severity. The valve-in-valve (ViV) procedure is particularly beneficial for patients with suboptimal positioning of their initial valve. In a study from the Italian registry involving 663 patients, 3.6% underwent a ViV procedure, and the outcomes at one-year follow-up showed comparable safety and efficacy to those who received a single valve [104]. A meta-analysis comparing the safety and efficacy of ViV-TAVI with redo-SAVR in failed bioprosthetic valves demonstrated that ViV-TAVI is a safe and feasible option for patients at high surgical risk [105]. This approach has proven to be highly effective in managing severe PVL, particularly in cases of undersized or malpositioned valves, with high procedural success rates and notable improvements in hemodynamic performance and symptom relief [13].
4. Percutaneous Closure Devices: Interventional closure of PVL after TAVI has been reported for both self-expandable and balloon-expandable prostheses. Percutaneous transcatheter closure using an Amplatzer Vascular Plug (AGA Medical Corp., Plymouth, MN, USA) can be considered when significant PVL persists, particularly in cases with a localized aortic regurgitation (AR) jet, due to severely calcified native valves, despite multiple attempts at balloon post-dilatation [106]. However, there are several potential risks associated with percutaneous transcatheter device closure of PVL after TAVI, including embolization of the transcatheter heart valve (THV), embolization of the closure device itself, and the risk of stroke. This minimally invasive approach is associated with significant improvements in both symptoms and hemodynamic performance, particularly for high-risk surgical candidates [107].
5. Emerging Techniques: New interventions, including arterio-arterial loops and imaging-guided approaches, are under investigation. These techniques aim to improve the accuracy and safety of PVL closure while minimizing procedural risks [107].

## 7. Future Directions

### 7.1. Device Innovations

Newer generation THVs and their delivery systems are designed to further reduce the occurrence of procedure-related complications, particularly PVL, and thus improve the procedural outcomes. There are two main focuses: ability to reposition the valve and optimization of the sealing with the native valve with possibly no orifices left between the prosthesis and the annulus where PVL may occur. Most of the new valve types have had very promising study results [108].

The Boston Lotus Valve (Boston Scientific, Natick, MA, USA) is a bioprosthetic valve made from bovine pericardial tissue mounted on a woven nitinol frame that expands as the stent shortens. It is repositionable and can be recaptured prior to full deployment. To mitigate PVL, it incorporates an adaptive seal at its lower end. In the REPRISE III randomized clinical trials (RCTs), which involved 912 patients (607 Lotus vs. 305 CoreValve), the Lotus Valve demonstrated lower PVL rates compared to CoreValve across all valve sizes. However, no difference in all-cause mortality was observed between the two groups [109].

The Medtronic Evolut R (Medtronic, Minneapolis, MN, USA) represents an evolution of the first-generation CoreValve. Its extended skirt enhances sealing to prevent PVL while maintaining the original CoreValve’s cell geometry for better frame adaptation to native anatomy. This valve is also recapturable and repositionable. Registry data comparing Evolut R to earlier-generation valves for transcatheter aortic valve implantation (TAVI) revealed improved short-term outcomes, with lower incidences of PVL and other complications [105].

The Edwards SAPIEN 3 (Edwards Lifesciences, Irvine, CA, USA) is a balloon-expandable prosthesis with a lower profile. It features inner and outer polyethylene terephthalate (PET) sealing skirts to reduce PVL. A non-randomized registry of 1592 intermediate- and high-risk patients undergoing TAVI with the SAPIEN 3 reported that at 30 days, 55.7% had less than mild PVL, 32.6% had mild, 8.2% had mild-to-moderate, and only 3.5% had moderate PVL. One-year all-cause mortality was 9.3%, with higher mortality among patients with moderate PVL. Interestingly, 73% of patients with moderate PVL at 30 days showed a reduction in PVL severity by one grade at one year [62].

The Symetis ACURATE Valve (Symetis, Lausanne, Switzerland) is a self-expanding porcine bioprosthetic valve mounted on a nitinol stent. It includes a sealing skirt at its lower crown for optimal sealing at the annulus level. A European multicenter registry of 1000 patients undergoing TAVI with the ACURATE neo transfemoral valve reported favorable outcomes at 30 days, with a mean effective orifice area of 1.77 ± 0.46 cm^2^, a mean pressure gradient of 8.4 ± 4.0 mmHg, and low PVL rates (only 4.1% of patients had more than mild PVL) [110].

Last but not least, a recent registry study revealed promising data regarding the safety and long-term effectiveness of percutaneous closure of paravalvular leak (PVL) after transcatheter aortic valve implantation (TAVI). Overall, 45 patients that underwent PVL closure with (64% male) with the Amplatzer Valvular Plug III and the Amplatzer Valvular Plug 4 were enrolled. The median age was 80 years (75–84). Among them, 67% and 33% had self-expanding and balloon-expandable valve implantations, respectively. Baseline post-TAVI PVL was severe in 67% of cases and moderate in the rest. The time from index TAVI to PVL closure procedure was 16.1 (8.7–34.8) months. Most patients were in NYHA Class III and IV (73%) before the procedure, and 40% had referred hospitalizations for heart failure between TAVI and the PVL closure procedure. Successful PVL closure was achieved in 94%, reducing regurgitation to ≤ mild in 91% and moderate in the rest. The incidence of severe adverse events was 11%. None of the patients died during the index hospitalization. During long-term follow-up (21.7 ± 16.2 months), the all-cause mortality rate was 14%, and patients presented improvement in functional status and a significant reduction in the rate of hospitalization for heart failure (from 40% to 6%) [28].

### 7.2. Imaging Advancements

Accurate imaging plays a pivotal role in reducing PVL by enabling precise procedural planning and intraoperative guidance. The Valve Academic Research Consortium-3 recommends echocardiography as the primary imaging method for the evaluation of PVL [111]. However, the integration of three-dimensional (3D) and real-time imaging technologies is expected to revolutionize TAVI workflows. Advanced imaging modalities such as 3D transesophageal echocardiography (TEE) and real-time computed tomography (CT) enable operators to visualize the aortic annulus, valve morphology, and surrounding structures with unprecedented detail, even in patients with difficult acoustic windows. This enhanced visualization aids in the accurate sizing and positioning of transcatheter heart valves, thereby minimizing the risk of PVL [62,112].

Furthermore, real-time imaging during valve deployment provides immediate feedback on the interaction between the prosthetic valve and native annular tissue. Techniques such as real-time fusion imaging—combining fluoroscopy and echocardiography—facilitate precise adjustments during deployment. These advancements not only enhance procedural safety but also improve the likelihood of achieving optimal valve-sealing outcomes.

### 7.3. Need for Standardization of PVL Assessment

Despite significant advancements in TAVI technology and procedural techniques, the lack of standardized methods for assessing PVL remains a critical challenge. Current approaches to PVL evaluation vary widely across institutions and practitioners, leading to inconsistencies in diagnosis, grading, and clinical decision-making. To address this issue, there is an urgent need for universally accepted definitions and standardized protocols for PVL assessment.

A comprehensive framework for PVL evaluation should incorporate multimodal imaging, including echocardiography, CT, and magnetic resonance imaging (MRI), to provide a holistic view of the leak’s severity and impact [28,112]. Grading systems must also be refined to account for variations in PVL’s clinical significance, considering factors such as location, circumferential extent, and hemodynamic consequences. Additionally, efforts should focus on developing automated or semi-automated tools to enhance the objectivity and reproducibility of PVL measurements.

Standardization is essential not only for clinical practice but also for research and device development. A unified approach would facilitate the comparison of outcomes across studies, enabling a more accurate evaluation of new devices and techniques aimed at reducing PVL. Ultimately, these efforts will support the broader goal of improving long-term outcomes for patients undergoing TAVI.

## 8. Limitations

This manuscript has several inherent limitations associated with its narrative review format. Primarily, it lacks the methodology and quantitative rigor of a formal systematic review and meta-analysis. Although we have incorporated comparative studies to discuss outcomes between various transcatheter heart valves, the evidence presented should be interpreted cautiously, acknowledging these methodological constraints. Future systematic reviews and meta-analyses, as long as RCTs are necessary to provide more definitive conclusions regarding the incidence, predictors, and clinical outcomes associated with PVL in patients undergoing TAVI.

## 9. Conclusions

Paravalvular leak (PVL) remains a significant challenge in the context of transcatheter aortic valve implantation (TAVI), despite remarkable advances in technology and procedural techniques. This review has comprehensively addressed the incidence, predictors, mechanisms, diagnostic modalities, and management strategies associated with PVL, highlighting its profound impact on patient outcomes and long-term prognoses. The advent of TAVI has revolutionized the management of severe aortic stenosis, offering a less invasive alternative to surgical aortic valve replacement (SAVR), particularly for high-risk populations. However, as TAVI indications extend to younger and lower-risk populations, addressing complications like PVL becomes paramount. PVL not only diminishes the hemodynamic benefits of TAVI but is also associated with increased morbidity and mortality, underscoring the need for continued innovation and vigilance.

The incidence of moderate-to-severe PVL has declined significantly due to enhancements in valve design, imaging techniques, and operator experience. Newer-generation valves, such as the Sapien 3 and Evolut Pro, have integrated features like sealing skirts and advanced deployment mechanisms, reducing PVL rates to below 5% in most cases. However, mild PVL remains prevalent, affecting approximately 20–30% of patients and contributing to long-term complications like left ventricular remodeling and heart failure.

Addressing PVL requires a multifaceted approach that begins with a thorough understanding of its predictors. Patient-specific factors, including annular size and shape, left ventricular ejection fraction, and calcification distribution, play critical roles. Device-specific factors, such as valve type and deployment depth, also significantly influence outcomes. The integration of advanced imaging modalities, such as 3D computed tomography (CT) and real-time transesophageal echocardiography (TEE), has enhanced pre-procedural planning and intra-procedural guidance, enabling more precise valve sizing and positioning.

Management strategies for PVL are tailored to its severity and clinical impact. Mild PVL may be managed conservatively, while moderate-to-severe cases often require intervention. Techniques such as percutaneous closure, post-deployment balloon dilation, and valve-in-valve implantation have demonstrated efficacy in reducing PVL severity and improving outcomes. Emerging technologies, including machine learning and computational fluid dynamics, hold promise for further optimizing PVL prediction and prevention.

Looking ahead, the focus must shift to further reducing even mild PVL, as its clinical significance becomes more apparent in younger and healthier populations with longer life expectancies. Innovations in valve design, such as repositionable and recapturable devices, and advancements in imaging techniques, including real-time fusion imaging, are expected to play pivotal roles. Moreover, the standardization of PVL assessment protocols is critical to ensuring consistent diagnosis and treatment across institutions.

In conclusion, while TAVI continues to evolve as a transformative therapy for aortic stenosis, addressing PVL remains essential for maximizing its long-term success. Collaborative efforts between clinicians, researchers, and industry stakeholders are needed to drive innovation, refine techniques, and improve patient outcomes. By prioritizing the mitigation of PVL, the TAVI community can ensure that this life-saving procedure reaches its full potential in improving the quality and longevity of patients’ lives.

## Figures and Tables

**Table 1:** Criteria for prosthetic aortic valve regurgitation *.

Three-Class Grading Scheme	None/Trace	Mild		Moderate		Severe
Five-Class Grading Scheme	None/Trace	Mild	Mild-Moderate	Moderate	Moderate-Severe	Severe
Doppler parameters (qualitative or semi-quantitative)						
Jet features						
Extensive/wide jet origin	Absent	Absent	Absent	Present	Present	Present
Multiple jets	Possible	Possible	Often present	Often present	Usually present	Usually present
Jet path visible along the stent	Absent	Absent	Possible	Often present	Usually present	Present
Proximal flow convergence visible	Absent	Absent	Absent	Possible	Often present	Often present
E/A ratio	<1.0	<1.0	<1.0	≥1.5	≥1.5	≥1.5
Vena contracta width (mm) (color Doppler)	Not quantifiable	<2	2 to <4	4 to <5	5 to <6	≥6
Vena contracta area (mm^2^) (3D color Doppler)	Not quantifiable	<5	5 to <10	10 to <20	20 to <30	≥30
Jet width at its origin (%LVOT diameter) (color Doppler)	Narrow (<5)	Narrow (5 to <15)	Intermediate (15 to <30)	Intermediate (30 to <45)	Large (45 to <60)	Large (≥60)
Jet density (CW Doppler)	Incomplete or faint	Incomplete or faint	Variable	Dense	Dense	Dense
Jet deceleration rate (PHT, ms) (CW Doppler)	Slow (>500)	Slow (>500)	Variable (200 to <500)	Variable (200 to <500)	Variable (200 to <500)	Steep (<200)
Diastolic flow reversal in proximal descending aorta (PW Doppler)	Absent	Absent or brief early diastolic	Intermediate	Intermediate	Holodiastolic (end-diastolic velocity 20 to <30 cm/s)	Holodiastolic (end-diastolic velocity ≥30 cm/s)
Circumferential extent of PVL (%) (color Doppler)	Not quantifiable	<5	5 to <10	10 to <20	20 to <30	≥30
Doppler parameters (quantitative)						
Regurgitant volume (mL/beat)	<15	<15	15 to <30	30 to <45	45 to <60	≥60
Regurgitant orifice area (mm^2^)	<5	<5	5 to <10	10 to <20	20 to <30	≥30
Regurgitant fraction (%)	<15	<15	15 to <30	30 to <40	40 to <50	≥50

* Adapted from Généreux et al. [62], Pibarot et al. [71], Ruiz et al. [63], and Zoghbi et al. [28]. 3D, 3-dimensional; LVOT, left ventricular outflow tract; CW, continuous wave; PHT, pressure half-time; PW, pulsed wave; PVL, paravalvular regurgitation.

**Table 2:** Invasive hemodynamic parameters.

Parameter	Formula	Cutoff for Significance
DD [81,83]	DAP − LVEDP	DD ≤ 32 mmHg highest predictive value for relevant PVL DD ≤ 18 mmHg predictor of 30-day and 1-year mortality
HR-DD or CHAI score [82]	(DD/heart rate) × 80	HR-DD or CHAI score < 25 mmHg/bpm (indicating ≥moderate PVL) predictor of 1-year mortality
ARI [84]	([DBP − LVEDP]/SBP) × 100	ARI < 25 predictor of 1-year mortality
ARI ratio [85,86,90]	Post-procedural ARI/pre-procedural ARI	ARI ratio < 0.60 predictor of 1-year mortality ARI ratio < 0.60 improves 1-year mortality prediction of post-TAVI ARI < 25 Higher predictive value than MSCT AVCS for PVL after TAVI requiring PD
DPTI [87]	[(area between aortic and LV diastolic pressure-time curves/diastolic duration)/SBP] × 100	DPTI ≤ 27.9 predictor of 1-year mortality
TIARI [88,89]	(DPTI/LV SPTI) × 100	TIAR index < 80 associated with ≥mild AR Higher TIARI is associated with better survival after TAVI

DD, diastolic delta; HR, heart rate; CHAI, composite heart-rate-adjusted hemodynamic-echocardiographic aortic insufficiency; ARI, aortic regurgitation index; DPTI, diastolic pressure-time integral; TIARI, time-integrated aortic regurgitation index; DAP, diastolic aortic pressure; LVEDP, left ventricular end-diastolic pressure; PVL, paravalvular leak; bpm, beats per minute; DBP, diastolic blood pressure; SBP, systolic blood pressure; MSCT, multi-slice computed tomography; AVC, aortic valve calcium; TAVI, transcatheter aortic valve implantation; PD, post-dilation; LV, left ventricle; SPTI, systolic pressure-time integral.
